# Design and Implementation of Real-Time Software Radio for Anti-Interference GPS/WAAS Sensors

**DOI:** 10.3390/s121013417

**Published:** 2012-10-01

**Authors:** Yu-Hsuan Chen, Jyh-Ching Juang, Jiwon Seo, Sherman Lo, Dennis M. Akos, David S. De Lorenzo, Per Enge

**Affiliations:** 1 Department of Aeronautics and Astronautics, Stanford University, 496 Lomita Mall, Stanford, CA 94305, USA; E-Mails: shinge@stanford.edu (Y.-H.C.); daedalus@stanford.edu (S.L.); dsd@stanford.edu (D.S.D.); penge@stanford.edu (P.E.); 2 Department of Electrical Engineering, National Cheng Kung University, 1 University Road, Tainan 70101, Taiwan; E-Mail: juang@mail.ncku.edu.tw; 3 School of Integrated Technology, Yonsei University, 162-1 Songdo-dong, Yeonsu-gu, Incheon 406-840, Korea; E-Mail: jiwon.seo@yonsei.ac.kr; 4 Department of Aerospace Engineering Sciences, University of Colorado, 1111 Engineering Drive, Boulder, CO 80309, USA; E-Mail: dma@colorado.edu

**Keywords:** Global Positioning System (GPS) and Wide Area Augmentation System (WAAS) sensor, software-defined radio, controlled reception pattern antenna (CRPA), Space-Time Adaptive Processing (STAP), radio frequency interference

## Abstract

Adaptive antenna array processing is widely known to provide significant anti-interference capabilities within a Global Navigation Satellite Systems (GNSS) receiver. A main challenge in the quest for such receiver architecture has always been the computational/processing requirements. Even more demanding would be to try and incorporate the flexibility of the Software-Defined Radio (SDR) design philosophy in such an implementation. This paper documents a feasible approach to a real-time SDR implementation of a beam-steered GNSS receiver and validates its performance. This research implements a real-time software receiver on a widely-available x86-based multi-core microprocessor to process four-element antenna array data streams sampled with 16-bit resolution. The software receiver is capable of 12 channels all-in-view Controlled Reception Pattern Antenna (CRPA) array processing capable of rejecting multiple interferers. Single Instruction Multiple Data (SIMD) instructions assembly coding and multithreaded programming, the key to such an implementation to reduce computational complexity, are fully documented within the paper. In conventional antenna array systems, receivers use the geometry of antennas and cable lengths known in advance. The documented CRPA implementation is architected to operate without extensive set-up and pre-calibration and leverages Space-Time Adaptive Processing (STAP) to provide adaptation in both the frequency and space domains. The validation component of the paper demonstrates that the developed software receiver operates in real time with live Global Positioning System (GPS) and Wide Area Augmentation System (WAAS) L1 C/A code signal. Further, interference rejection capabilities of the implementation are also demonstrated using multiple synthetic interferers which are added to the live data stream.

## 1. Introduction

Global Navigation Satellite Systems (GNSS) signals are relatively weak and thus vulnerable to deliberate or unintentional interference [1]. An electronically-steered antenna array system provides an effective approach to mitigate interference by controlling the reception pattern and steering beams/nulls. As a result, so called Controlled Reception Pattern Antenna (CRPA) arrays have been deployed by organizations such as the U.S. military which seeks high levels of interference rejection. However, the increased cost and computational complexity has not been acceptable for the civil commercial market thus far. This paper demonstrates a low cost CRPA implementation developed and implemented using the flexible software radio approach.

In the literature, CRPA receivers have been implemented by different approaches. Williams *et al.* [2] used a four-element antenna array and a CRPA system to implement spatial nulling adaptive array. The CRPA system used an analogue approach to combine the signals and adopt a correlation feedback to derive the weight vector. Konovaltsev *et al.* [3] implemented a MATLAB-based software receiver to assess the performance of the beamforming algorithms and found the steering vectors by Direction of Arrival (DoA) algorithms. Li *et al.* [4] proposed a beamforming architecture for real Intermediate Frequency (IF) signals and calculated optimum weights from given GPS almanac. Heckler *et al.* [5] developed a four-element antenna array and front-ends for dual-band L1/L5 signals. They also implemented a MATLAB-based software receiver for field test of adaptive beamforming algorithms with directions of satellites calculated from DoA estimation. These MATLAB-based software receivers do not have real-time capabilities. Arribas *et al.* [6] implemented a real-time hardware and software platform which is capable of digital beamforming. They used a Field Programmable Gate Array (FPGA) for digital beamforming up to eight antenna elements and delivered the resulting spatially-filtered digital signals to a Personal Computer (PC) by Ethernet bus. The signal can be stored for post-processing or processed by a real-time software receiver [7,8]. De Lorenzo *et al.* [9] describe various approaches for beamforming (pre- and post-correlation) in a post-processing software radio architecture. Seo *et al.* [10] use a quad-core Central Processing Unit (CPU) coupled with a Graphics Processing Unit (GPU) to implement an all-in-view CRPA software receiver. However, no previous publication has documented the design or implementation of an all-in-view pre-correlation real-time CRPA software receiver for GNSS sensors using CPU alone.

The bit-wise parallel software correlation algorithm [11] has the potential to enable a real-time software receiver if digitized GNSS signals have 2-bit resolution. Yet 2-bit resolution does not provide sufficient dynamic range when the CRPA design is most valuable—in the presence of high power interferer. Thus, the bit-wise parallel software correlation algorithm is not sufficient for any practical CRPA implementation which would demand 8 or more bits of sampling resolution.

This paper completely documents a four-element CRPA software receiver implementation capable of processing 16-bit complex samples generating up to twelve beamformed channels for the Global Positioning System (GPS) and Wide Area Augmentation System (WAAS) L1 Coarse/Acquisition (C/A) code signals in real time.

Conventionally, antenna array system receivers perform CRPA processing with the geometry of the antennas and cable lengths known in advance. In this software receiver, the implemented algorithm allows CRPA operation without such prior knowledge. Instead, the carrier phase differences between satellite tracking channels are used as the constraints of adaptive beamforming algorithm as a simplistic model for Minimum Variance Distortionless Response (MVDR) [12] pre-correlation beamforming. Further, the implementation includes Space-Time Adaptive Processing (STAP) [13] which provides best interference rejection performance but has highest computational complexity among interference rejection techniques [14].

In order to achieve real-time capability, the most computationally expensive functions are programmed in x86 assembly using Single Instruction Multiple Data (SIMD) instructions. We build on a structure of SIMD parallel programming [15] and include SIMD assembly code as a real-time implementation example since it is the fundamental enabler of such an implementation. Multi-threaded programming, again fully documented, is leveraged to fully exploit the multi-core resources of the processor [16]. The execution flow is designed to distribute the tasks across multiple cores. For reducing the operations of the software correlator, local replicas of code and carrier at zero-phase are generated and stored prior to execution. A software technique to correlate the IF data with local replica for whole code period without crossing data bit transition is developed and described.

The implementation is validated using actual IF data streams. The signal processing, positioning and beamforming are accomplished by software approach and shown to function in real time. By adding synthetic interference to the collected data, the only practical test option given the protected nature of the GPS L1 frequency band, two test scenarios are used to demonstrate the interference rejection performance of the CRPA software receiver. The first interference scenario is with high Interference-to-Signal ratio (J/S). The second scenario is with multiple different interferer types from different directions. The results are illustrated by angle-frequency responses.

The key contributions of this paper are summarized as follows: (a) this is the first published work which not only demonstrates a real-time all-in-view CRPA software receiver running on a CPU but describes the implementation in significant detail with actual code examples critical to the architecture; (b) This work leverages a mechanism to perform “blind” beamforming (without prior knowledge or calibration). This mechanism is implemented by realizing up to 60 tracking channels (12 tracking channels for each antenna element of the four-element array and 12 tracking channels for composite beamformed signal). (c) This work provides an approach to estimate Radio Frequency (RF) chain/cabling bias upon startup. This information is not necessary for beamforming in our approach but it can be useful to illustrate the functionality of the receiver. Based on this bias information, which is used to generate a simplified antenna model, angle-frequency responses are calculated accurately to show the functionality of the implemented STAP in the spatial and temporal domains in the presence of multiple interferers with different bandwidths.

The paper proceeds as follows: first, the beamforming algorithm implemented in the software receiver is introduced and its mathematical properties are outlined. Then, the mechanism used to build the steering vectors without prior calibration is documented. The architecture of the software receiver, including hardware and software components, is explained in detail. In order to validate the implementation and the interference rejection performance, the software receiver is tested with live GPS/WAAS data and synthetic interferers. The results demonstrate the performance of the software receiver under multiple interferers in the spatial and temporal domains.

## 2. Beamforming Algorithm Used in the Software Receiver

The primary goal of CRPA is to enhance the carrier-to-noise ratio (C/N_0_) of desired signal and reject the interferers. Digital beamforming approaches are used to implement CRPA by combining the signals of an antenna array. Various signal combination approaches are well documented in the literature, which include frequency-processing, spatial-processing, STAP [13] and Space-Frequency Adaptive Processing (SFAP) [17]. Frequency-processing is primarily against narrowband interference. Spatial-processing is efficient against both broadband and narrowband interferers, but it can cancel only *N*-1 interferers, where *N* is the number of elements in the antenna array. STAP or SFAP places nulls both in the frequency and spatial domain and can cancel more than *N*-1 interferers if some interferers are narrowband. Despite their benefit, the computational complexity of STAP or SFAP is high, so implementing them in a real-time software receiver is challenging. Several algorithms have been proposed to calculate weights for STAP or SFAP. Some algorithms optimize certain conditions with known signal structure of the desired signal such as maximum signal-to-interference ratio, minimum mean square error and minimum output power. Other algorithms do not need prior knowledge of signal structure but minimize output power to certain constraints such as MVDR [12] and Constrained Least Mean-Squares [18]. The constraints can be set to form a beam in the direction of satellite or steer a null in the direction of interference. The steering vector associated with the direction of satellite can be obtained from either satellite ephemeris and array calibration information or even carrier phase differences between elements of an antenna array can be leveraged as a simplified model.

**Figure 1.**
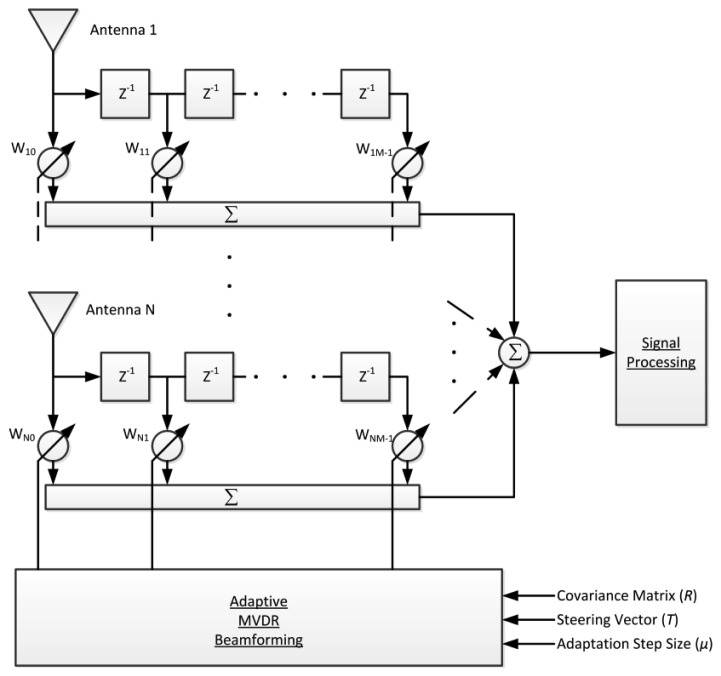
Architecture of STAP with adaptive MVDR beamforming algorithm.

In order to be able to reject multiple interferers with different bandwidths, we adopt the STAP with adaptive MVDR beamforming algorithm [19] to implement CRPA shown in Figure 1. After down-conversion to zero IF and digitization, the IF signal of each antenna is complex and denoted as *S_n_*[*k*]. Each signal is then multiplied by complex weights *w_n_* and summed over all antennas to make a composite signal as follows:

(1) $$\begin{array}{l} y[k]=\sum_{n=1}^{N}\sum_{m=0}^{M-1}w_{\text{nm}}s_{n}[k-m]=W^{H}S[k]\\ W={\left[w_{10}\;w_{20}\;\cdots \;w_{N0}\;w_{11}\;\cdots w_{N1}\;\cdots \;w_{N0}\;\cdots \;w_{N(M-1)}\right]}^{T}\\ S[k]={\left[\underline{s}[k]\;\underline{s}[k-1]\;\cdots \;\underline{s}[k-(M-1)]\right]}^{T}\\ \underline{s}[k]=\left[s_{1}[k]\;s_{2}[k]\;\cdots \;s_{N}[k]\right] \end{array}$$

where *N* is the number of elements in the antenna array. *M* is the number of time taps. *s_n_*[*k* − *m*] is the signal from *n*^th^ antenna element and *m*^th^ time tap. *w_nm_* is the weight associated to the *n*^th^ antenna and *m*^th^ tap.

The MVDR algorithm minimizes the output power and constraints the gain of the direction of desired signal to unity as follows [18]:

(2) $$\begin{array}{c} \text{minimize}{\left|y\right|}^{2}=W^{H}\text{RW}\\ \text{subject to}\;W^{H}C=1 \end{array}$$

where *R* is the covariance matrix of input signals and *C* is the constraint vector toward the target satellite. The covariance matrix *R* is estimated by computing sample covariance matrix with assuming the sample mean is zero as follows:

(3) $$\begin{array}{c} R[k]=\left[\begin{array}{ccccccc} r_{1_{0}1_{0}} & r_{1_{0}1_{1}} & \cdots & r_{1_{0}1_{M-1}} & r_{1_{0}2_{0}} & \cdots & r_{1_{0}N_{M-1}}\\ r_{1_{1}1_{0}} & r_{1_{1}1_{1}} & & & & & \\ \vdots & & \ddots & & & & \\ r_{1_{M-1}1_{0}} & & & r_{1_{M-1}1_{M-1}} & & & \vdots \\ r_{2_{1}1_{0}} & & & & r_{2_{0}2_{0}} & & \\ & & & & & \ddots & \\ r_{N_{M-1}1_{0}} & & & \cdots & & & r_{N_{M-1}N_{M-1}} \end{array}\right]\\ r_{a_{k}b_{i}}=\frac{1}{N}\sum_{t=0}^{N_{s}-1}s_{a}[t-k]\cdot \bar{s_{b}[t-l]} \end{array}$$

where *N_s_* is the number of samples to compute the covariance matrix. The overline sign is defined as complex conjugation. For each entity in the *R* matrix, $r_{ab}=\bar{r_{ba}}$ *a* and *b* stand for the index of antenna elements.

The constraint vector *C* is composed of a steering vector *T* as the first to *N*^th^ elements and zeros as the remaining (*M* − 1)*N* elements:

(4) $$\begin{array}{l} C={\left[t_{1}\;t_{2}\;\cdots \;t_{N}\;\underset{(M-1)N}{\underset{︸}{0\;\cdots \;0}}\right]}^{T}\\ T={\left[t_{1}\;t_{2}\;\cdots \;t_{N}\right]}^{T} \end{array}$$

**Figure 2.**
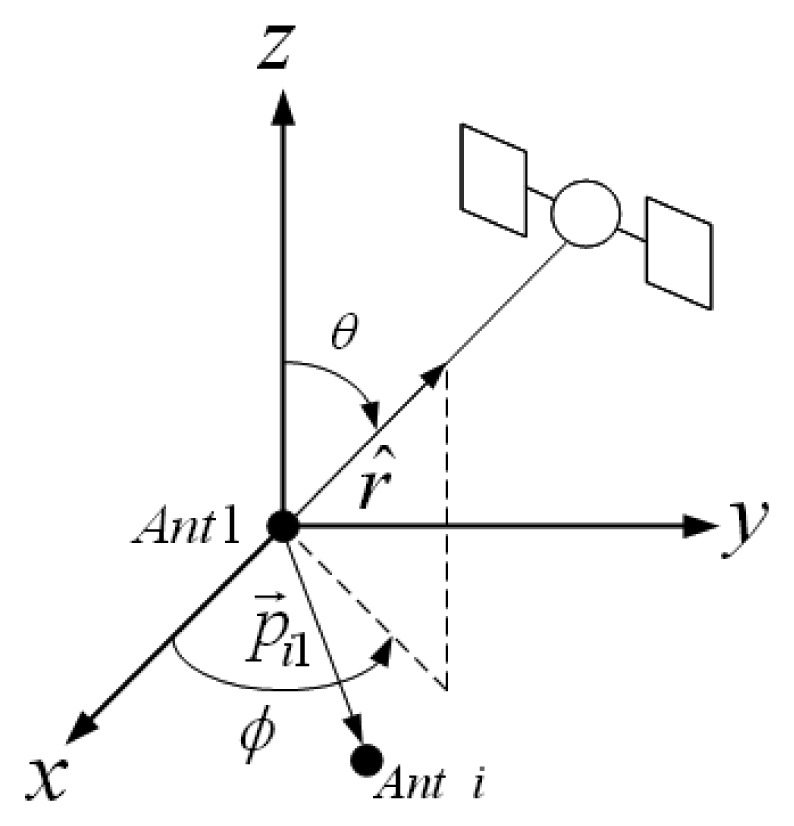
Antenna geometry and direction of a satellite showing calculation of $\Delta \varphi_{i1}^{l}$.

Traditionally, the signal of the first antenna element is set as the reference and its component in the steering vector is set to 1. The other components are set as phase shifts relative to the reference antenna, which are represented by:

(5) $$t_{i}=\exp(-j(\Delta \varphi_{i1}^{l}+\Delta \gamma_{i1}))$$

where $\Delta \varphi_{i1}^{l}$ is the phase difference based on antenna geometry and the direction of desired satellite. Δ*γ_i_*_1_ is the phase resulting from the difference of cabling and RF chain including down converter and digitization among elements of antenna array. $\Delta \varphi_{i1}^{l}$ can be calculated as:

(6) $$\Delta \varphi_{i1}^{l}=\frac{2\pi {\vec{p}}_{i1}\cdot {\hat{r}}^{l}(\phi ,\theta )}{\lambda }$$

where *p⃗_i_*_1_ is the baseline vector of the *i*^th^ antenna and *r̂^l^*(*φ*, *θ*) is the unit vector to satellite *l* shown in the Figure 2.

The baseline vector *p⃗_i_*_1_ is known from the array geometry, and *r̂^l^*(*φ*, *θ*) can be obtained through positioning. Only Δ*γ_i_*_1_ needs to be re-calibrated whenever any part of hardware of antenna array is changed. From the derivation in [18], the solution of Equation (2) is:

(7) $$W=R^{-1}C{\left(C^{T}R^{-1}C\right)}^{-1}$$

Two matrix inversions are needed to compute in Equation (7). Alternatively, the Frost algorithm [18] derived an adaptive approach which iteratively updates the weight:

(8) $$\begin{array}{c} W[0]=\frac{1}{N}C\\ W[i+1]=\left(I-\frac{1}{N}CC^{T}\right)(I-\mu R[i])W[i]+\frac{1}{N}C \end{array}$$

where *μ* is the adaptation step size which can be constant or variable related to covariance matrix. *i* is the iteration count. In our software receiver, *μ* is calculated by:

(9) $$\mu =\overset{\alpha }{\;}/\underset{\text{trace}(R)}{\;}$$

where *α* is a constant dependent on the number of entries in the *R* matrix.

## 3. Obtaining the Steering Vector without Prior Calibration

**Figure 3.**
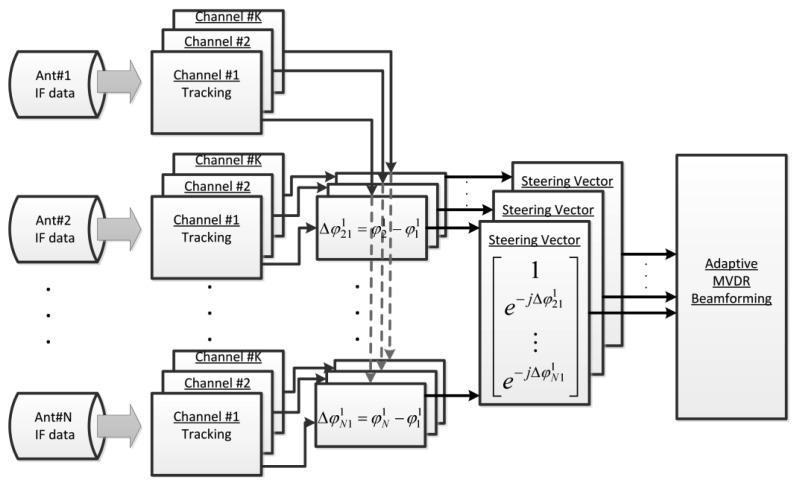
Mechanism to obtain the steering vectors in the software receiver.

For adaptive MVDR beamforming, obtaining the steering vector is the key to implementing a CRPA. However, as mentioned in the previous section, some parameters of the steering vector need to be calibrated. Direction vectors toward satellites can be obtained after positioning. A software receiver has the flexibility of implementing multiple channels to track multi-antenna signals separately. Integrated Carrier Phase (ICP) is one of the tracking outputs of the phase lock loop. The ICP is often used to smooth code pseudorange for improving accuracy of positioning. In our software receiver, ICP differences between different antenna elements are taken to build the steering vectors instead of deriving the vectors from the azimuths/elevations of the satellites and the baseline vectors of antenna elements [20]. Figure 3 shows the block diagram of the approach used to obtain the steering vector in our software receiver. This approach enables us to perform beamforming without a prior calibration.

However, if ICP cannot be extracted from individual antenna elements due to weak signal, this technique does not work. If the ICP cannot be extracted due to interference, the steering vector is kept as previously calculated value. For our implementation, it is assumed that the receiver is initiated in interference free conditions.

The IF data of each antenna is processed by *K* tracking channels (*K* = 12 in our case) in which each channel is assigned to track one satellite. Each satellite is tracked by *N* channels (*N* = 4 in our case) assigned to *N* antenna elements. Once the *N* channels assigned to a given satellite are simultaneously tracked, the ICPs are initialized by filtered phases of the in-phase (I) and quadrature (Q) components of correlator outputs. Then, the ICPs are continuously integrated and the phase differences between the reference antenna and other antennas are computed at runtime. The differences of ICPs between different antennas provide elements of the steering vector. This phase difference, which is the same as $\Delta \varphi_{i1}^{l}+\Delta \gamma_{i1}$ in Equation (5), can also be used to calculate the cabling/RF chain part Δ*γ_i_*_1_ because $\Delta \varphi_{i1}^{l}$ is obtained after positioning.

## 4. Architecture of Software Receiver

The developed CRPA real-time software receiver runs on a PC platform and uses IF datasets as input where the detailed description of the data collection hardware is presented in [7]. It currently operates on GPS and WAAS L1 C/A code signals and supports four-element antenna array.

**Figure 4.**
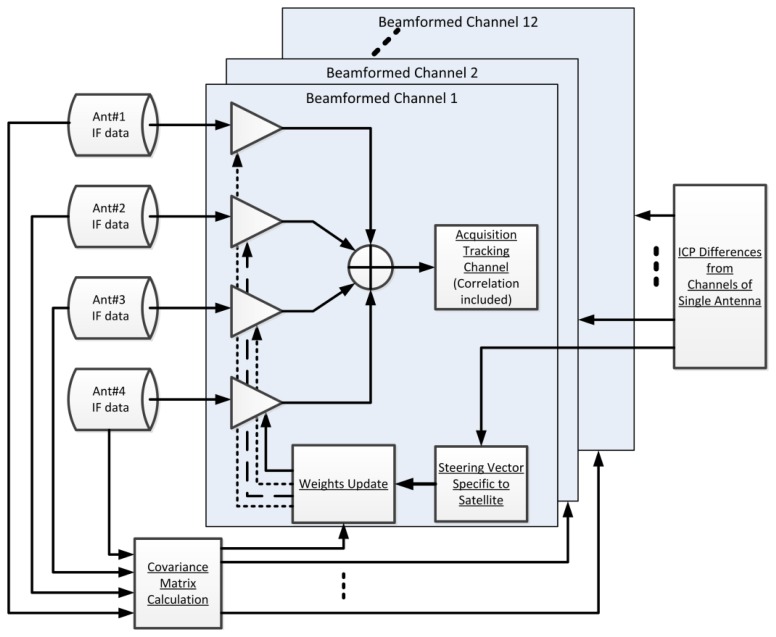
Beamforming architecture for steering 12 beams simultaneously using the MVDR algorithm.

There are a total of 60 channels, of which 48 channels are used to process data from four antennas (12 channels for each antenna element) and are dedicated to obtain the ICP differences only. The other 12 channels are used to process the beamformed composite signal using the pre-correlation beamforming approach shown in Figure 4. The STAP algorithm, which is capable of rejecting more than *N*-1 interferers is implemented in the pre-correlation approach. Each beamformed channel uses one set of weights to steer a beam toward a satellite. The weights are updated by adaptive MVDR algorithm. The steering vectors, which are inputs to the weight update, are obtained from ICP differences between tracking channels as described in the previous section. Hence, the software receiver can form beams before positioning in the pre-correlation sense.

The weight update rate of MVDR algorithm is 1 kHz for adapting highly dynamic interferers. The current platform can achieve real-time performance with up to five time taps for STAP processing. There exists a tradeoff between the number of antenna elements, tracking channels, time taps, and weight update rates. Given the four-element antenna array, our software receiver can reject at most three broadband interferers. It can reject more than three interferers if some of them are narrowband nature. Positioning is dedicated to the beamformed composite signals. In order to illustrate the interference rejection performance, the cabling/RF chain biases need to be calculated after positioning. Then, the biases combined with weights are used to calculate the accurate angle-frequency response which provides a coarse visualization of the outputs of STAP given the electrical layout of the individual elements.

### 4.1. Software Architecture

The software is developed using Microsoft Visual Studio under 64-bit version of Microsoft Windows 7. Most source codes are programmed using C++. The functionalities with high computational complexity, such as correlation operation and covariance matrix calculation, are programmed by assembly. The component list of the software is in Table 1, and detailed explanations of the components are given in the following subsections.

#### 4.1.1. Automatic Gain Control

Software receiver processing starts from reading the IF data from the disk. The MVDR algorithm works efficiently when the data received from different antenna gains/RF chain has similar distribution. Hence, IF data from each antenna is amplified by a gain term which is managed by an automatic gain control function. The objective of the gain control is to equalize the different noise power of all elements of antenna array caused by different RF chains/antenna gains. The gain is updated iteratively by finding a maximum value across one ms data as follows:

(10) $$\begin{array}{l} \text{MAX}=\overset{N_{s}}{\underset{i=1}{\max}}\left|s_{i}\right|\\ g=(2^{N_{b}}-1)/\text{MAX}\\ G[k+1]=G[k]+\beta (g-G[k]) \end{array}$$

where *s_i_* is *i*^th^ digitized sample of the IF signal, *N_s_* is the number of samples in one ms, *N_b_* is the number of bits for representing data, and *β* is updating step.

#### 4.1.2. Software Correlator

**Figure 6.**
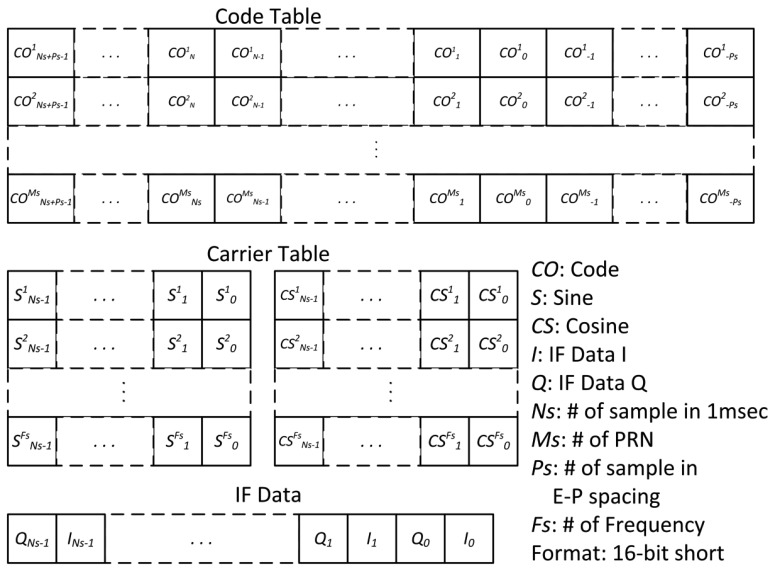
Structure of code table, carrier table, and IF data used in the software correlator.

In the software correlator, the code and carrier of IF signals are wiped off. The 16-bit complex data (I/Q) are correlated with local code and carrier replica. Due to its high computational complexity, the software correlator is programmed in assembly using SIMD instructions. Based on the SIMD library correlator in Hecker and Garrison [15], an assembly code architecture optimized for the specific code and carrier tables are created prior to execution. The structures of code, carrier table and input IF data are depicted in Figure 6. These tables are formatted as 16-bit short integers. The code table is made by *N_s_* + 2*P_s_* phase for each PRN code where *P_s_* is the number of samples between Early-to-Prompt spacing and *N_s_* is the number of samples in one ms. The carrier table is divided by sine and cosine tables and starts from zero phase for each Doppler frequency. I and Q of IF data are bundled together to make a pair. The correlation operation is done between IF data with the nearest sample to zero carrier phase and local Early/Prompt/Late replicas according to code phase measurement.

The correlation operation with complex IF data is performed as follows:

(11) $$\begin{array}{l} \underline{CI}+j\underline{CQ}=\sum_{k=0}^{N_{s}-1}CO_{k}(CS_{k}+jS_{k})(I_{k}+jQ_{k})\\ \underline{CI}=\sum_{k=0}^{N_{s}-1}CS_{k}\times CO_{k}I_{k}-S_{k}\times CO_{k}Q_{k}=\sum_{k=0}^{N_{s}-1}CI_{k}\\ \underline{CQ}=\sum_{k=0}^{N_{s}-1}CS_{k}\times CO_{k}Q_{k}-S_{k}\times CO_{k}I_{k}=\sum_{k=0}^{N_{s}-1}CQ_{k} \end{array}$$

where *CI̲* and *CQ̲* are the real part and imaginary part of correlator output, respectively. The other terms are defined in Figure 6.

**Figure 7.**
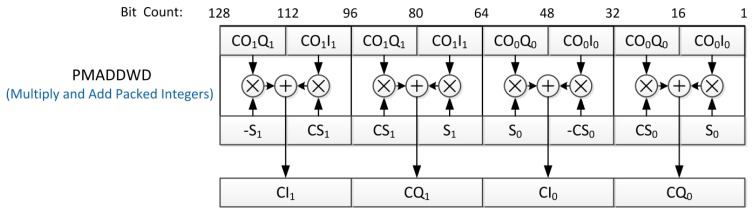
Complex data format for executing PMADDWD instruction and operations inside PMADDWD.

Using SIMD instructions and 128-bit-wide SSE registers [21], one can simultaneously operate on eight 16-bit-wide words by a single instruction. For example, in the step 3 in Table 2, the complex data format for executing the parallel multiply-and-add (PMADDWD) instruction is depicted in Figure 7. In total, eight multiplications and four additions are performed in one instruction cycle for processing two complex IF samples, so it would reduce computational load approximately 12x.

**Figure 8.**
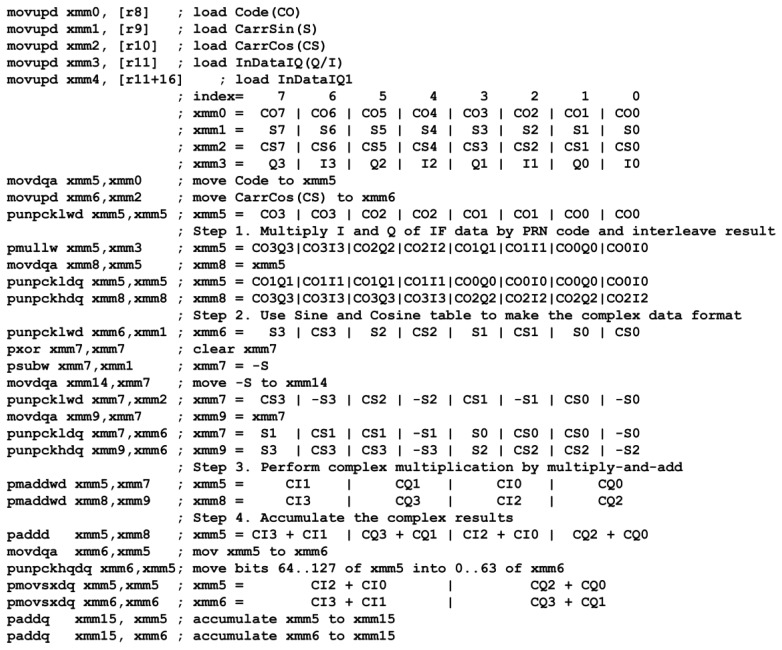
Assembly code example of software correlator using SIMD instructions.

An assembly code of the software correlator, which is compatible to SSE2, SSE3, and SSE4.1 [21], is programmed and shown in Figure 8 as an example. This code is built upon the library in Heckler and Garrison [15]. Additionally, instructions from the new version of SSE such as interleaving and parallel addition are incorporated to further increase performance. This code implements the inner part of the summation in Equation (11) and follows the operation procedure in Table 2. Four complex samples are processed in parallel within 30 instructions. This code is the key component to process 60 tracking channels in real time and thus its complete description has been included.

**Figure 9.**
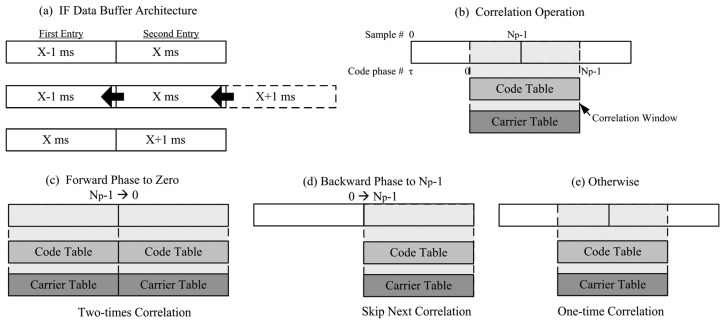
(**a**) IF data buffer architecture (**b**) correlation window (**c**-**e**) three cases for correlation operation which avoid performing correlation with data bit transition.

In order to avoid performing correlation across data bit transition point, two ms long buffers are used in parallel and one ms segment is selected to perform the correlation. The selection approach is to use the code phase measurement to set the starting sample of correlation window. Then, this zero-code-phase segment is correlated with zero-phase PRN code and carrier tables for whole code period. Figure 9(a) shows the architecture of IF data buffer. When new data comes in, the oldest data will emerge, the second entry is shifted to the first entry, and the new data is loaded to the second entry. Figure 9(b) shows the correlation window which is set by current code phase measurement. However, code phase would shift forward or backward due to Doppler frequency. A strategy is adopted to account for code phase movement. According to the direction of code phase movement, there are three possible cases as shown in Figure 9(c–e) and listed as follows

1. Forward phase movement to zero (phase Np-1in 1st entry and phase 0 in 2nd entry): perform correlation two times for the first and the second buffers.
2. Backward phase movement to Np-1 (phase 0 in 1st entry and phase Np-1 in 2nd entry): perform a correlation for the second buffer and skip next correlation.
3. Others: perform a correlation across two entries.

#### 4.1.3. Acquisition/Tracking and Positioning

These functions adopt open source codes from Greenberg and Ebinuma [22] by replacing its interface which controls hardware correlator by our software correlator. Additionally, the WAAS signal processing and Viterbi decoder for messages are added to support positioning with the WAAS GEO. The structure of carrier tracking loop of software receiver for the specific formats of code and carrier table is described in Juang and Chen [23]. And, the phase compensation for carrier wipe-off is implemented using a technique in Ledvina *et al.* [11].

#### 4.1.4. ICP Initialization and Calculation of Differential ICP

The ICP is obtained by integrating IF carrier frequency over time as follows:

(12) $$\varphi_{p}[k]=\int_{0}^{t_{k}}\omega dt+\phi_{0}\approx \sum_{t=0}^{k-1}\omega [i]T_{I}+\phi_{0}$$

where *φ*_0_ is the initial phase, *ω* is the Doppler frequency and *T_1_* is the PRN code period which is one msfor C/A code. Juang and Chen [23] derived the phase of correlator outputs as:

(13) $$\theta_{c}[k]=\text{atan}2(CQ[k],CI[k])=\frac{(\omega_{R}-\omega )T_{I}}{2}-\omega t_{k-1}-\phi_{0}$$

where *θ_c_*[*k*] is the phase at *k* ms based on the correlator outputs and the rounded Doppler frequency, *ω_R_*. The correlator outputs, *CI*[*k*] and *CQ*[*k*], are calculated using the carrier table which has limited frequency resolution. *ω_R_* is the frequency after rounding the Doppler frequency *ω* to the nearest frequency grid of the carrier table. Hence, the compensated phase of incoming signal at time *t_k_* can be obtained by:

(14) $$\varphi_{p}[k]=\omega t_{k-1}+\phi_{0}+\omega T_{I}=-\theta_{c}[k]+\frac{(\omega_{R}+\omega )T_{I}}{2}$$

The effect from the rounded frequency in *θ_c_*[*k*] is compensated in *φ_p_*[*k*] by Equation (14). Since this compensated phase is noisy, an averaging filter is used to reduce the noise for estimating initial phase *φ*_0_:

(15) $$\begin{array}{c} \phi_{p}[1]=\varphi_{p}[1]\\ \phi_{p}[k]=\frac{k}{M_{a}}\left(\mathrm{mod}\left(\phi_{p}[k-1]+\omega [k-1]\cdot T_{I},2\pi \right)\right)+\left(1-\frac{k}{M_{a}}\right)\varphi_{p}[k],2\leq k\leq M_{a} \end{array}$$

where *φ_p_*[*k*] is the averaged phase with less noise, and *M_a_* is the number of ms to perform the initialization. *k* denotes *k*^th^ ms. After the initialization process, the residual of *φ_p_*[*M_a_*] is set as the initial phase *φ*_0_ as follows:

(16) $$\phi_{0}=\mathrm{mod}(\phi_{p}[M_{a}],2\pi )$$

Once the initial phase is obtained, the ICP is calculated every ms by adding *ω* [*i*]*T_I_* as in Equation (12). In order to build the steering vector, differential ICPs between antennas are calculated for every channel. Then, the differential ICPs of several ms are averaged to obtain the steering vectors as follows:

(17) $$\begin{array}{l} \Delta \phi_{n1}^{l}=\sum_{i=1}^{L}\left(\phi_{n}^{l}[i]-\phi_{1}^{l}[i]\right)\\ t_{n}^{l}=\exp(-j\Delta \phi_{n1}^{l}) \end{array}$$

where *L* is the number of ms to average the differential ICP. *l* and *n* stand for *l*^th^ channel and *n*^th^ antenna. $t_{n}^{l}$ is *l*^th^ channel and *n*^th^ antenna element of the steering vector.

#### 4.1.5. Adaptive MVDR Beamforming

The adaptive MVDR beamforming function starts from computing the covariance matrix. The covariance matrix in Equation (3) is a Hermitian matrix, so only the elements of the upper triangular part need to be computed and the remaining elements can be obtained by the conjugation operation. The computational complexity of Equation (3) is significant. Indeed, in the four antennas and five time taps case, there are 210 elements to be computed. The computational load for each element is *N_s_* complex 16-bit multiply-and-add operations. With the sampling rate of 4 MHz, 4,000 multiply-and-add operations per element need to be completed within 1 ms. Hence, two approaches are adopted to expedite the computation. First, multithreaded programming approach is used to assign operations to multiple threads for exploiting the resources of multi-core CPU. Second, SIMD assembly programming is leveraged to accelerate the complex multiply-and-add operations. The procedure to compute covariance matrix initializes with the loading of the complex IF samples from both *a*^th^ and *b*^th^ antenna element. Then, the multiply-and-add and accumulation operations, as the 3rd and 4th steps in Table 2, are performed. After computing the covariance matrix, the adaptation step size is decided by Equation (9). Lastly, the adaptive procedure in Equation (8) for each channel is performed to update its weight using calculated covariance matrix, steering vector, and adaptation step size.

#### 4.1.6. Weight and Sum

**Figure 10.**
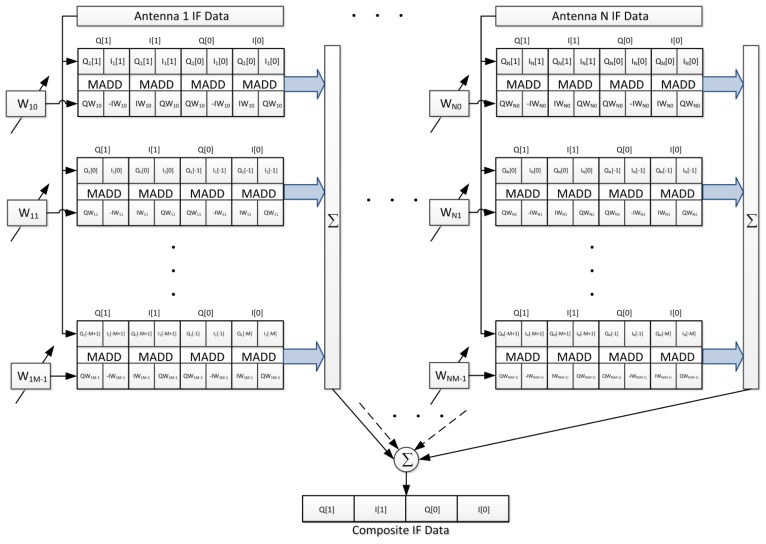
Architecture of weight and sum including data formatting and used SIMD instructions.

After a set of updated weights is obtained from adaptive MVDR beamforming function, the weight-and-sum function performs the combination of IF data as Equation (1). The results are sent to software correlator as composite IF data input. Assembly code using SIMD instructions implements the weight-and-sum operation by an architecture shown in Figure 10. By arranging the complex data format, one multiply-and-add (MADD) instruction processes the complex multiplication of two samples and outputs a product including I and Q components. In the end, all products are summed as composite IF data.

#### 4.1.7. Bias Calculation

Even though this approach can perform beamforming without any calibration/modeling, the RF chain/cabling bias information is useful to show the performance of interference rejection of the CRPA receiver by angle-frequency response. Hence, these biases are estimated in the software receiver. This bias information is not necessary for the beamforming process as previously explained. The bias estimation process starts from obtaining the differential ICPs and unit vectors to satellites through positioning. The differential ICP for *k*^th^ satellite between *i*^th^ and first antenna is given by:

(18) $$\Delta \varphi_{i1}^{k}=\frac{2\pi {\vec{p}}_{i}\cdot {\hat{r}}^{k}}{\lambda }+\Delta \gamma_{i}+N_{i}^{k}+{ɛ}_{i}^{k}$$

where $N_{i}^{k}$ is the integer associated to $\Delta \varphi_{i1}^{k}$, and ${ɛ}_{i}^{k}$ is the phase error. Then, with known baseline vectors, the fractional part of bias can be estimated by:

(19) $$fr(\Delta \gamma_{i})=\frac{1}{K}\sum_{k=1}^{K}\mathrm{mod}(\Delta \varphi_{i1}^{k}-\frac{2\pi {\vec{p}}_{i}\cdot {\hat{r}}^{k}}{\lambda },2\pi )$$

where *K* is the number of channels. Only the fractional part of bias is required to calculate the angle-frequency response because integer part of bias can be ignored inside exponential function. This bias estimation does not consider the biases from mutual coupling and phase-center variations.

#### 4.1.8. Calculation of Angle-Frequency Response

After the STAP processing, antenna array response in the direction of *φ*, *θ* in Figure 2 and at the frequency *f* is given by:

(20) $$GP(\phi ,\theta ,f)=\sum_{n=1}^{N}\sum_{m=0}^{M-1}w_{nm}\times \exp\left[j\left(\frac{2\pi {\vec{p}}_{n}\cdot \hat{r}(\phi ,\theta )}{\lambda }+\Delta \gamma_{n}-2\pi fmT_{s}\right)\right]$$

where *T_s_* is sampling time. There are three dimensions of the response, two for the spatial domain and one for the spectral domain. If one would like to show the spatial response such as the gain pattern, then the frequency is fixed and gain values of two direction angles are shown on the polar plot. The gain pattern is useful to show the spatial performance of beamforming because directional beams and nulls are clearly illustrated. As another representation of array response, assuming that one of the directional angles of interference is known, one fixes one directional angle and shows the gain of the other directional angle *versus* frequency, which is called angle-frequency response. The angle-frequency response is used to simultaneously show the spatial and spectral performance of STAP where narrowband notch filter and broadband null steering are simultaneously illustrated.

### 4.2. Multi-Threaded Programming and Real-Time Execution Flow

**Figure 11.**
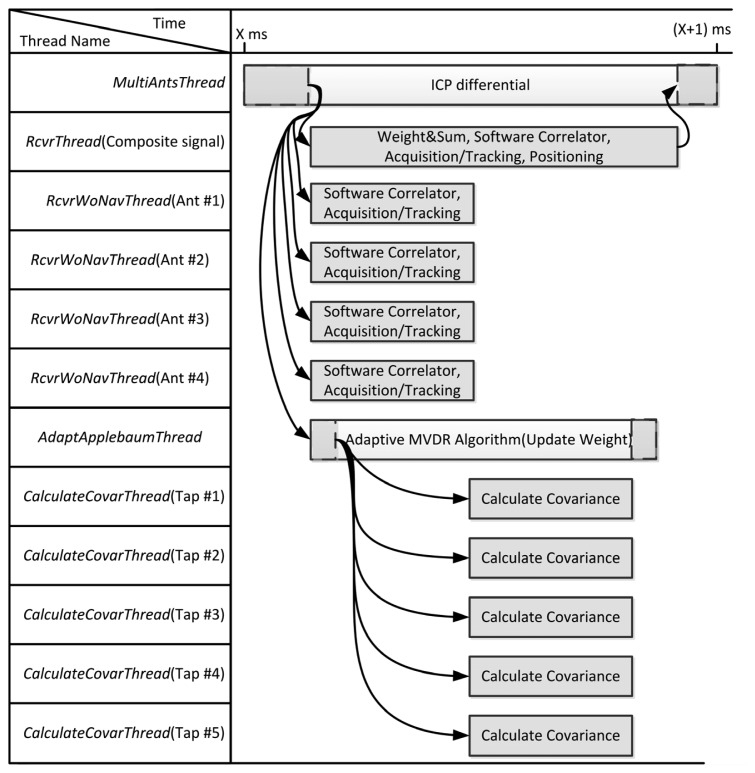
Execution flow of the software receiver within 1 ms. Multithread programming is used to make the software capable of real-time execution.

The system program schedules the threads to execute the software receiver for fully exploiting the resource of CPU. Figure 11 shows the execution flow plan for the receiver using 4 antennas and 5 time taps. To achieve real-time capability, the execution flow must be finished within 1 ms for 1 ms input signals. The *MultiAntsThread* masters the whole flow to synchronize the threads using event objects. At first, *MultiAntsThread* sets events to receiver threads and an adaptation thread for initiating the individual receiver operation and weight updating. For the composite beamformed signal, *RcvrThread* performs the weight-and-sum using weight from previous iteration, software correlator, acquisition/tracking and positioning. The positioning result will be given by this thread. For each antenna element, each *RcvrWoNavThread* performs software correlation and acquisition/tracking only. The ICPs are measured by these threads. For adaptation, the *AdaptApplebaumThread* first sets events to multiple *CalculateCovarThread* to calculate the elements of the covariance matrix, and then perform the MVDR algorithm to update the weights. Finally, the *MultiAntsThread* uses the differential ICPs to build the steering vectors.

## 5. Interference Rejection Performance of CRPA Software Receiver

**Figure 12.**
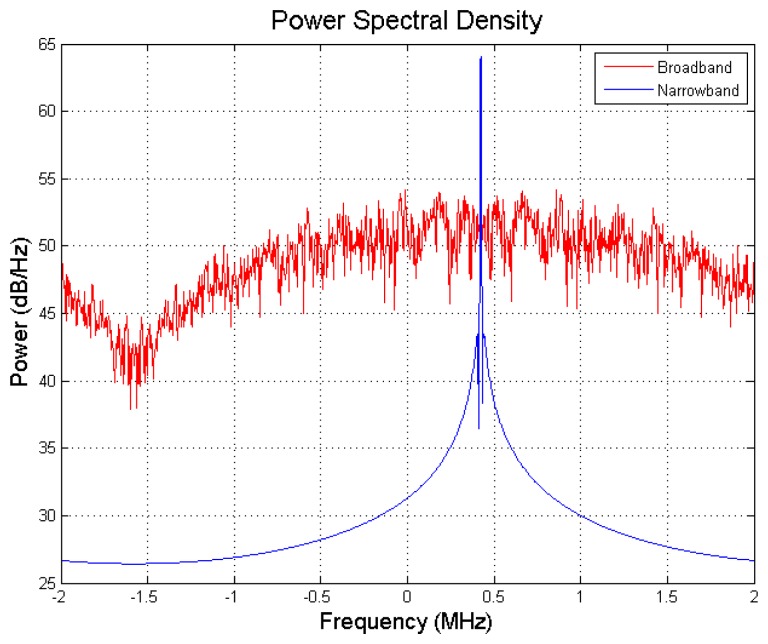
Power spectral density of broadband and narrowband interferers.

A dataset is collected by the signal collection hardware [24] in open field (low multipath environment) to examine the interference rejection performance of the CRPA software receiver. The RF front-end of signal collection hardware is a Universal Software Radio Peripheral (USRP) [25] with a Bitshark USRP Broadband configurable RF receiver (BURX) daughter board [26]. Its electrical details are as follows. Sampling rate is set as 4 MHz. Intermediate frequency bandwidth is set as 4 MHz. Noise figure is 6.8 dB. Gain is set as 20 dB. Bits per sample at ADC output are 14 bits for both I/Q. For injecting the interference, a MATLAB script is written to generate the synthetic interferers and combine them with original dataset. The direction and signal specification of interferers can be specified in the script. Two scenarios are built. In the first scenario, a single, broadband and high J/S interferer is injected. In the second scenario, multiple broadband/narrowband with moderate J/S interferers are injected. The power spectral density of broadband and narrowband interferers is shown in Figure 12. The 3dB-bandwidth of broadband interferer is 2 MHz and narrowband interferer is a continuous sinusoidal wave.

### 5.1. Single Interferer with High J/S

**Figure 13.**
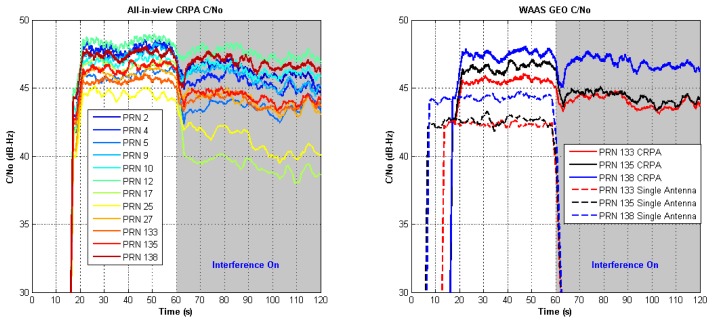
(**left**) C/N_0_ of all-in-view CRPA channels. (**right**) C/N_0_ of three WAAS GEO channels (CRPA and single antenna cases).

**Figure 14.**
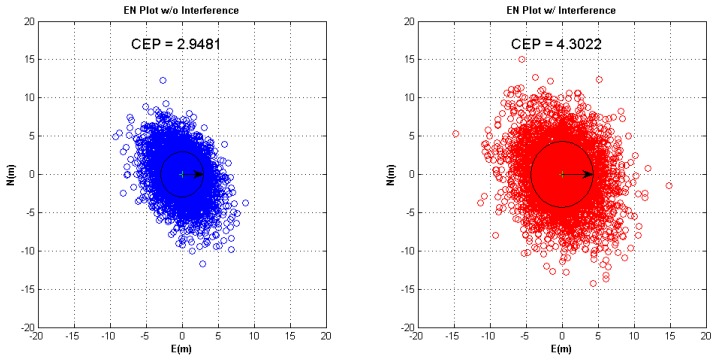
(**left**) Positioning results of the software receiver without interference. (**right**) Positioning results of the software receiver with interference.

A single interferer specified by 40 dB J/S, 30° elevation angle, and 60° azimuth angle is simulated to examine the software receiver performance. The interferer is added into dataset starting from 60th second. Figure 13 (left) shows the filtered C/N_0_ of all-in-view CRPA. All channels are still in tracking when high J/S interferer is injected. Figure 13 (right) shows the C/N_0_ of three WAAS GEOs to compare performance of CRPA and single antenna. Before injecting the interferer, there is about 5 dB gain in C/N_0_ of CRPA over single antenna. When the interferer is injected, the C/N_0_ of all WAAS GEO channels in the single antenna lose lock, but the channels of CRPA continue tracking without losing more than 5 dB of the original C/N_0_. Figure 14 shows Earth-North (EN) plots with and without interference. The interferer affects the positioning result with worse Circular Error Probable (CEP) because the interferer increases the noise level.

### 5.2. Multiple Interferers

In order to examine the rejection capabilities of the software receiver to multiple interferers, six interferers are injected to the data set as indicated by the sequence shown in Table 3.

**Figure 15.**
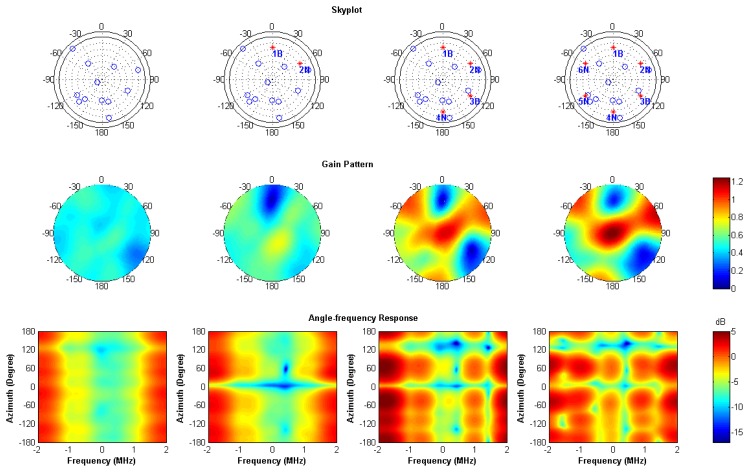
Sky plots and angle-frequency responses of four cases with injected interferers marked by red dots in the sky plots.

Using a four-element array, the maximum number of broadband interferers that can be nulled using spatial adaptation is three. In this scenario, two broadband and four narrowband interferers are injected to examine the performance of STAP. Figure 15 shows the sky plots, antenna gain patterns and angle-frequency responses of four cases. The antenna gain patterns and angle-frequency responses are averaged over all the channels for showing common nulls in the plots. The represented gains are obtained by fixing the elevation angle as 30°.

**Figure 16.**
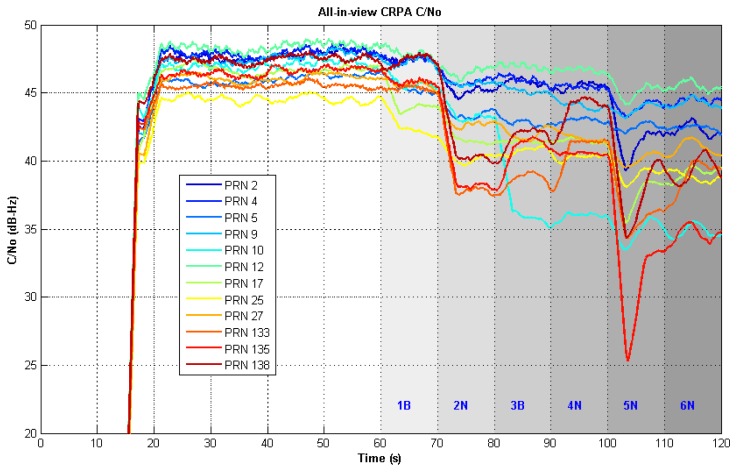
All-in-view CRPA channels C/N_0_ under two broadband and four narrowband interferers

Each column in Figure 15 stands for each interferer case. The interferers are shown by red dots in the sky plot. In the first column, there is no interferer, so no obvious null or notch is present. In the second column, when one broadband and one narrowband interferers are injected, one null appear at the azimuth angle 0° in the gain pattern and one narrowband notch appear at the frequency of 0.42 MHz in the angle-frequency response. In the end, total four narrowband and two broadband interferers are injected. There are two nulls in the direction of broadband interferers and four notches at the frequency of the narrowband interferers. Note that adaptation using the time taps makes notches in the frequency domain instead of steering nulls in the spatial domain. It is the reason why the number of interferers which can be rejected is more than the number of elements in the antenna array minus one. Figure 16 shows all-in-view CRPA C/N_0_ when adding multiple interferers. All channels continue tracking throughout the injecting sequence of interferers. This validates the CRPA software receiver is capable of mitigating multiple interferers.

## 6. Conclusions

This paper describes the implementation of a software receiver for GPS/WAAS L1 C/A code signals to demonstrate the feasibility of CRPA technology for civil applications. The developed approach performs pre-correlation beamforming without any prior calibration. The architecture of STAP is constructed in a software approach to achieve a CRPA. The optimum weights obtained by adaptive MVDR algorithm provide the interference rejection performance capable of rejecting multiple interferers.

The components of software receiver are implemented and constructed efficiently using the SIMD assembly and C/C++ multithreaded programming. In particular, the SIMD assembly code has been developed and optimized to achieve a fast correlation process of high resolution (16 bits) samples and is fully documented within the paper.

The software receiver implementation is validated, demonstrating it can perform all-in-view pre-correlation beamforming up to twelve channels in real-time. The angle-frequency response calculation is also included in the software to illustrate the interference rejection performance of the receiver. The results of our experiments demonstrate interference rejection capability in the presence of a single higher power broadband interference with 40 dB J/S or six lower power broadband/narrowband interferers with 30 dB J/S.

In the next generation of x86 CPUs, a new instruction set called Advanced Vector Extensions (AVX) will be incorporated which will have the capacity to further expedite the computation of the vector operations. The width of the SIMD register will be 256 bits, two times wider than that of the current registers. This would reduce the computational load by two times. Moreover, the AVX instruction set introduces a three-operand format where two source operands and one destination operand. This would decrease the number of instructions for moving data. Using the AVX instruction set, the software receiver would gain the ability to process wider bandwidth signals such as GPS/WAAS L5 signals in real time, which require a higher sampling rate. The structure of our software receiver is flexible to extend to GPS/WAAS L5 processing with minimal modifications. The Federal Aviation Administration (FAA) Alternative Position Navigation and Timing (APNT) study is interested in the use of CRPA with the WAAS L5 signal for robust time transfer under GPS interference [24].

## Figures and Tables

**Figure 5.**
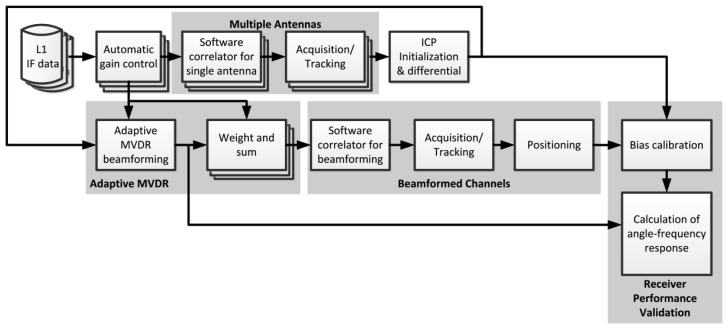
Flowchart of the software from reading IF data to validating receiver performance.

**Table 1.** Component list of the software architecture including short descriptions and how often each component is called.

**Component**	**Description**	**Frequency**
Automatic gain control	Equalize noise power of all elements of antenna array to full 16-bit representation. Hand-coded assembly.	Call right after reading every 1 ms data from solid state drive.

Software correlator	Perform 16-bit complex data correlation by software approach. Hand-coded assembly.	every 1 ms

Acquisition/Tracking and Positioning	Signal processing and positioning function for GPS/WAAS satellites.	every 1 ms for acquisition/tracking, every 100 ms for positioning

ICP initialization and calculation of the differential ICP	Perform the initialization and difference calculation of ICP for determining the steering vectors.	every 1 ms

Adaptive MVDR beamforming-Covariance calculation-Weight updating	Implement the adaptive MVDR beamforming including covariance matrix calculation and weight updating.Hand-coded assembly for covariance calculation.	every 1 ms

Weight and sum	Perform the signal combination as Equation (1).	every 1 ms
	Hand-coded assembly.	

Bias calculation for receiver performance validation	Perform the calculation of cabling/RF chain bias.	One time after first positioning

Calculation of angle-frequency response for performance validation	Perform the calculation of angle-frequency response.	every 5 s

System Program	Schedule threads to achieve real-time capability.	every 1 ms

**Table 2.** Procedure to implement software correlator with complex IF data for a single sample.

**Step**	**Description**	**Operations**
1.	Multiply I and Q of IF data by PRN code and interleave the result	1 multiplications
		2 interleaving

2.	Arrange Sine and Cosine tables in complex data format	4 interleaving

3.	Perform complex multiplication by multiply-and-add	2 multiply-and-add

4.	Accumulate the complex results	3 additions
		1 interleaving

**Table 3.** Injecting sequence of multiple interferers.

**Sequence**	**Interferer Type**	**J/S**	**IF Frequency**	**Direction**
1B	Broadband	30 dB	0.42 MHz	Azimuth: 0° Elevation: 30°
2N	Narrowband	30 dB	0.42 MHz	Azimuth: 60° Elevation: 30°
3B	Broadband	30 dB	0.42 MHz	Azimuth: 120° Elevation: 30°
4N	Narrowband	30 dB	1.42 MHz	Azimuth: 180° Elevation: 30°
5N	Narrowband	30 dB	−1.58 MHz	Azimuth: −120° Elevation: 30°
6N	Narrowband	30 dB	−0.58 MHz	Azimuth:−60° Elevation: 30°
